# Concentrations of Radiocesium in Local Foods Collected in Kawauchi Village after the Accident at the Fukushima Dai-ichi Nuclear Power Station

**DOI:** 10.1038/srep28470

**Published:** 2016-06-23

**Authors:** Makiko Orita, Kanami Nakashima, Naomi Hayashida, Yuuko Endo, Shunichi Yamashita, Noboru Takamura

**Affiliations:** 1 Department of Global Health, Medicine, and Welfare, Atomic Bomb Disease Institute, Nagasaki University, Nagasaki 8528523, Japan; 2 Division of Strategic Collaborative Research, Center for Promotion of Collaborative Research on Radiation and Environment Health Effects, Atomic Bomb Disease Institute, Nagasaki University, Nagasaki 8528523, Japan; 3 Kawauchi Municipal Government, Fukushima 9791201, Japan; 4 Department of Radiation Medical Sciences, Atomic Bomb Disease Institute, Nagasaki University, Nagasaki 8528523, Japan

## Abstract

We evaluated the current concentrations of radiocesium in local foods collected in Kawauchi Village, which is located less than 30 km from Fukushima Daiichi Nuclear Power Station, to minimize public anxiety regarding internal radiation exposure through the consumption of locally produced foods after the 2011 Fukushima accident. The number of samples exceeding the regulatory radiocesium limit (100 Bq/kg for general foods) was five out of 4,080 vegetables (0.1%), 652 of 1,986 (32.8%) among edible wild plants and fungi, and eight of 647 (1.2%) in fruits. Our study confirmed that the internal radiation doses of ingesting these foods are acceptably low compared to the public dose limit, ranging from 24.4 to 42.7 μSv for males and from 21.7 to 43.4 μSv for females, although the potential for radiation exposure still exists. Long-term comprehensive follow-up should take place to clarify trends in radiocesium concentrations in local foods and the committed effective doses found in Fukushima-area residents. By constructing a system that allows residents to access information on radiocesium concentration in foods, a risk communication model between specialists and residents could be developed in the recovery phase after the Fukushima accident.

On March 11, 2011, a massive earthquake struck the east coast of Japan. The earthquake, combined with the resulting tsunami, triggered a severe nuclear accident at the Fukushima Daiichi Nuclear Power Station (FDNPS)^1^. As a result, large amounts of radionuclides, including iodine-131 (^131^I), cesium-134 (^134^Cs) and cesium-137 (^137^Cs) were released into the atmosphere^2^. The United Nations Scientific Committee on the Effects of Atomic Radiation estimated the total release of ^131^I, ^134^Cs, and ^137^Cs at 120.0, 9.0, and 8.8 petabecqueral (PBq), respectively^2^.

Radiocesium (mainly ^134^Cs and ^137^Cs) is the predominant contributor to radiation exposure after the FDNPS accident because it has a relatively long half-life—^134^Cs has a half-life of 2.06 years and ^137^Cs 30.2 years—high transferability, and wide distribution in the environment^3^. Because of these relatively long half-lives, residents have expressed concerns about whether ^134^Cs and ^137^Cs may remain on the surface of soils in the fields for extended period time, and consequently be found in the plants growing on that land. Cesium (Cs) is an alkaline with chemical properties similar to potassium (K), which is an essential element for plants. Higher levels of K in the growth media are known to reduce plants’ uptake of Cs^4^.

Kawauchi Village, Fukushima Prefecture, is located within a 30 km-radius from FDNPS. After the May 2011 accident at FDNPS, almost all residents were evacuated outside the village (Fig. 1). On January 31, 2012, the mayor of the village declared that residents who lived at least 20 km away from FDNPS could return to their homes because the Japanese Prime Minister had declared that the reactors had achieved a state of cold shutdown in December 2011 and that radiation doses were found to be at comparatively low levels^5^. Since this declaration, the village office has been working steadily towards reconstruction. However, even though four years have now passed since return was authorized (and five since the accident), only about 60% of pre-accident population has returned. One reason that some residents have chosen not to return to the village is anxiety regarding radiation exposure^6^, especially the risks of internal exposure through the consumption of locally produced foods^7^. Recently, we evaluated the radiocesium concentrations in wild fungi collected at Kawauchi Village and found that radiocesium is often detectable^8^. On the other hand, radiocesium concentrations in other local samples should be also evaluated for food safety policy in Fukushima. Therefore, we examined the current concentrations of radiocesium in local food samples collected in the village and evaluated the internal effective doses of local residents.

## Results

The summary of radioactive contaminants in the surveyed foods is shown in Table 1. The number of samples exceeding the radiocesium regulatory limit (100 Bq/kg for general foods) is five (0.1%) for vegetables, 652 (32.8%) for edible wild plants and fungi, and eight (1.2%) for fruits.

The distribution of radiocesium in vegetables, edible wild plants and fungi, and fruits in Kawauchi Village is shown in Table 2. The radiocesium concentrations for all the samples reflected an extremely wide range in every season. In autumn, for example, concentrations of radiocesium in edible wild plants and fungi ranged from <20 to 2,754 Bq/kg for ^134^Cs and <23 to 8,487 Bq/kg for ^137^Cs.

**Figure 2:**
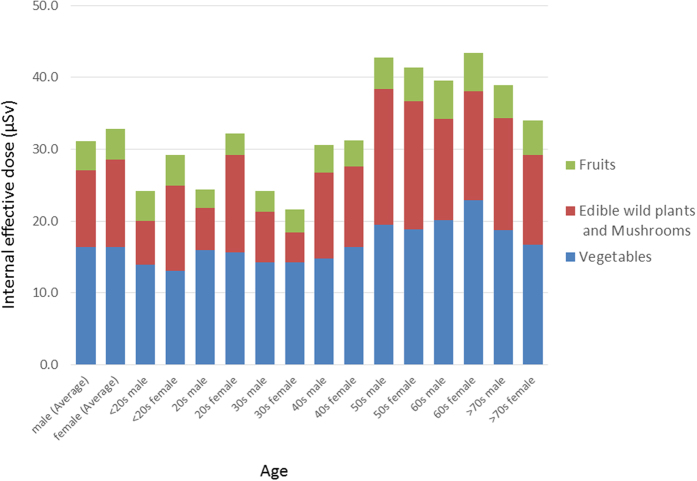
Internal effective doses due to radiocesium in Kawauchi Village, Fukushima Prefecture.

The committed effective doses from these agricultural products are summarized in Table 3. The committed effective doses ranged from 2.61 to 7.08 μSv for males and from 2.94 to 7.61 μSv for females, and from 1.88 to 5.75 μSv for male children and from 1.95 to 5.15 μSv for female children due to the ingestion of vegetables. The ingestion of edible wild plants and fungi led to ranges from 0.12 to 11.94 μSv for males and from 0.17 to 10.15 μSv for females and from 0.04 to 4.57 μSv for male children and from 0.50 to 7.77 μSv for female children. Finally, eating fruits led to a range of 0.25 to 1.65 μSv for males and from 0.50 to 1.65 μSv for females and from 0.55 to 1.64 μSv for male children and from 0.59 to 1.61 μSv for female children. The committed effective doses ranged from 24.4 to 42.7 μSv for all males and from 21.7 to 43.4 μSv for all females due to ingestion of the three food groups combined (Fig. 2).

## Discussion

The present study shows that radiocesium exceeding the current regulation value of radionuclides for foods (>100 Bq/kg) was detected in five of 4,080 (0.1%) vegetables and eight of 647 (1.2%) fruits collected in Kawauchi Village. Immediately after the accident, the Government of Japan established provisional regulation values for ^131^I (300 Bq/kg for drinking water and milk and 2,000 Bq/kg for vegetables) and ^134^Cs and ^137^Cs (200 Bq/kg for drinking water and milk and 500 Bq/kg for vegetables, grains, meats, fish, and eggs), and issued an order to restrict the shipment of food products exceeding these provisional values. Soon after, ^131^I exceeding provisional levels was detected in fresh cow milk, cultivated green leafy vegetables, and tapwater^9^, and foods that exceeded the provisional values were removed from the market^8^,^10^,^11^.

Five years have passed since the accident, and the concentration of radionuclides in food has decreased overall^12^. The Ministry of Health, Labour and Welfare (MHLW) has monitored radiocesium in foods since March 2011, using Ge semiconductor detectors or NaI scintillation detectors. They collected 16,712 vegetables and 3,302 fruits in Fukushima and its surrounding prefecture between April 2014 and March 2015, demonstrating that radiocesium exceeding the regulation values was not detected^13^.

In Kawauchi Village, the decontamination of residential areas and agricultural land has been underway since mid-2011, in accordance with the technical guidelines for decontamination developed by the Ministry of the Environment^14^. The top 5 cm of soil in which radiocesium was concentrated has either been completely removed or mixed with non-contaminated subsurface soil during tillage of agricultural lands. Forests within a 20-m radius from residential houses and agricultural lands have been decontaminated though the removal of branches, fallen leaves, and leaf mold^14^. Owing to these steps, substantial amounts of radiocesium have been removed from the area, contributing to the decrease of ambient dose rates. The current ambient dose of Kawauchi Village is 0.085 μSv/hour (May 5, 2016), whereas the highest ambient dose during the accident was 1.5 μSv/hour (March 15, 2011). Previously, we had evaluated the external radiation doses of residents who had temporally stayed in areas within a 20-km zone of the village and had estimated that the median annual dose was 1350 μSv/year^15^. These results suggest that the external exposure from living in Kawauchi Village is limited.

Our results showed that the radiocesium concentrations in edible wild plants and fungi were notably higher than in fruits and vegetables. Hoshi *et al*. reported that children consuming fungi showed a high ^137^Cs body burden near the plant^16^. It is well known that after the accident at the Chernobyl Nuclear Power Plant, wild fungi could be an important contributor to ^137^Cs body burdens, due to their capacity to accumulate ^137^Cs, and that certain population groups showed relatively high internal exposure due to intakes of such foodstuffs^17^. Since the accident at FDNPS, decontamination in the forests beyond a 20-m radius from residential houses has not been conducted^10^, leading to the relatively high concentrations of radiocesium in edible wild plants and fungi.

In this study, we showed that the lifetime committed effective doses ranged from 24.4 to 42.7 μSv for all males and from 21.7 to 43.4 μSv for all females, indicating that the health risk from internal exposure in residents through ingesting agricultural produce is extremely low. We had previously evaluated the committed effective doses from local agricultural samples in Kawauchi Village from May 2012 to March 2013, showing that the estimated doses ranged from 18 to 44 μSv/y for males and from 20 to 48 μSv/y for females, respectively^6^. Harada *et al*. also evaluated dietary exposure to radiocesium from a food-duplicate survey in three areas—Kawauchi Village, Tamano, and Haramachi—located between 20 and 50 km from FDNPS in 2012, reporting that the estimated internal dose rates from dietary intake of radiocesium averaged 5.8, 9.0, and 8.8 μSv/y in Kawauchi, Tamano, and Haramachi, respectively, while the estimated committed effective doses did not exceed the regulation values (<10^3^ μSv/y)^18^. These results show that the risk of internal exposure in Kawauchi Village residents is low since the initial phase of the accident.

Following the Chernobyl accident, it is well known that there was a seasonal difference in Cs body burden due to the seasonal change in diet and that the concentration of Cs body burden was higher in autumn; these results suggest that residents may have consumed products derived from the contaminated forests^17^. In this study, the seasonal change of radiocesium concentrations was observed only in “Edible wild plants and fungi” (Table 2). In spring and summer, most samples of this category are edible wild plants, but in autumn and winter, most samples are fungi. This difference in sample types causes the seasonal change in this category.

Although relatively high proportions of fish (mainly char), game (mainly wild boars), and crops (mainly chestnut) were above the regulatory limit, we excluded them from the analysis for committed effective doses since they were not actually consumed in the village.

Although residents who returned to the area after the accident had a higher likelihood of consuming locally produced vegetables than the general Japanese population, the food supply in those farming areas did not increase exposure to radiocesium because municipalities in Fukushima Prefecture routinely screen food products intended for human consumption.

The present study has several limitations. Our study was conducted only in Kawauchi Village, which might have led to sampling bias. Also, we could not evaluate analytical uncertainties since we had measured each sample only once. We used the average daily intake data of fungi and other foods issued before the accident (2009–2010), but the intake amount of such foods might decrease due to anxiety about the internal radiation exposure in Fukushima. Further comprehensive analyses with detailed reports on all areas around FDNPS are needed.

In conclusion, our study confirmed that the internal radiation doses of ingesting foods are acceptably low compared to the public dose limit, although the potential for radiation exposure still exists. Attention should be paid when consuming foods harvested from forests in order to avoid unnecessary chronic internal exposure. Moreover, long-term comprehensive follow-up should be undertaken to clarify trends in radiocesium concentrations in locally produced foods and the committed effective doses of residents in areas around FDNPS. By constructing a system that enables residents to access information on radiocesium concentration in foods, a risk communication model between specialists and residents could be developed in the recovery phase after the Fukushima accident.

## Materials and Methods

Local food samples were collected in Kawauchi Village. A radioactive contaminant survey for foods produced or collected in the village has been carried out by personnel at the village office since May 1, 2012. For this survey, 7,668 food samples produced or collected in the village were collected between April 2013 and December 2014. Of the total collected, 6,713 samples (4,080 vegetables, 1,986 edible wild plants and fungi, and 647 fruit samples) were selected for the current study, because these three items are major dietary components consumed by the residents and thus can be expected to contribute substantially to their internal radiation exposure. In this study, “vegetables” was defined as being grown and harvested from fields, and “wild plants and fungi” were natively grown in the wild. Wild fungi samples collected in this study were completely different samples from which we analyzed in the previous study^8^. The others, such as wild boars, chestnut, pheasants, and river fish, were also collected mainly for the investigation of radioactivity levels, but these samples were excluded from the analysis for committed effective doses because they were not actually consumed.

After preparation, local food samples (approximately 1000 g fresh) were put into plastic containers made of acrylic acid resin to measure the concentrations of ^134^Cs and ^137^Cs concentrations using an NaI detector (Canberra, CAN-OSP-NAI, AREVA NC Inc., Meridien) coupled to a multichannel analyzer (Genie 2000, Canberra Japan KK., Tokyo, Japan) for 1,800 s. The resolution of the instrument was <7.5% on 661.64 keV for ^137^Cs. ICRP.

The committed effective doses caused by the consumption of vegetables, edible wild plants and fungi and fruits were estimated from the radioactive fission product concentration, using the following formula:


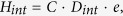


where *C* is the median activity concentration of detected radiocesium (Bq/kg fresh), *D*_*int*_ is the dose conversion coefficient for children’s intake (ages 0 to 19, 1.3 × 10^−5^−3.0 × 10^−5^  mSv/Bq for ^134^Cs and 9.6 × 10^−6^−2.2 × 10^−5^ mSv/Bq for ^137^Cs) and for adult intake (age 20 and older, 1.9 × 10^−5^ mSv/Bq for ^134^Cs and 1.4 × 10^−5^  mSv/Bq for ^137^Cs^19^, and *e* is the average daily intake data (g/day) for age, gender, and season in areas around FDNPS, as issued by the Environmental Radioactivity Monitoring Center of Fukushima, Fukushima Prefecture, Japan in 2009–2010^20^. We evaluated the committed effective doses using the following age groups: ages 0 to 12 months, 1 year to 3 years, 3 years to 8 years, 8 years to 13 years, 13 years to 18 years, and adults. In adults, we performed the calculation depending on the age classification and based on the average intake of food for every 10-year division. However, in children, we calculated the minimum and maximum values of the committed effective dose based on the average intake of 20 years to cover all age categories of children because we did not have the data for the average intake of food groups in the 19-years-or-less category.

In this study, we applied the recommendation of the Global Environment Monitoring System-Food Contamination Monitoring and Assessment Programme from the World Health Organization (GEMS/food of WHO), which the Japanese Ministry of Health, Labour and Welfare applied for the establishment of new standard limits for radionuclides in food^21^,^22^. In this recommendation, it is outlined that when the detection limit is not shown, 5 Bq/kg should be applied to food groups for which the ratio of “ND” (not detected) is not less than 80%; 10 Bq/kg should be applied to food groups for which the ratio of ND is 60% to 80%; and 20 Bq/kg should be applied to all others. Based on this recommendation, we calculated the committed effective doses, and the values under the radiocesium detection limits were assumed to have activity concentrations corresponding to 5 Bq/kg for vegetables and fruits and the detection limit itself (20 Bq/kg) for edible wild plants and fungi.

## Additional Information

**How to cite this article**: Orita, M. *et al*. Concentrations of Radiocesium in Local Foods Collected in Kawauchi Village after the Accident at the Fukushima Dai-ichi Nuclear Power Station. *Sci. Rep.* **6**, 28470; doi: 10.1038/srep28470 (2016).

## Figures and Tables

**Figure 1:**
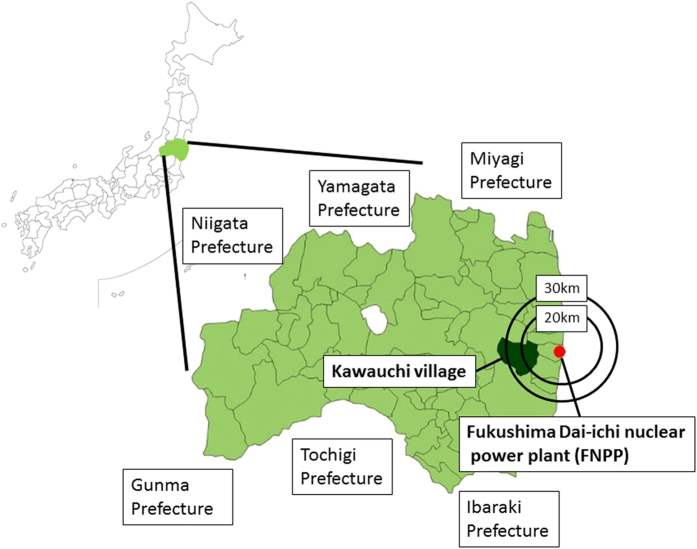
Location of Kawauchi Village, Fukushima Prefecture. The first author (M.O.) created the map using the software (Zenrin Electric Map Z i17^®^, ZENRIN CO., LTD., Tokyo, Japan. http://www.zenrin.co.jp/product/gis/zmap/zmaptown.html).

**Table 1:** Summary of the radioactive contaminants survey for foods.

Food type	Number of samples in the analysis	Number in excess of the regulatory limit for radiocesium (100 Bq/kg for general foods)	Percentage above the regulatory limit for radiocesium
Vegetables	4,080	5	0.1
Edible wild pants and mushrooms	1,986	652	32.8
Fruits	647	8	1.2
Fish^a^	36	14	38.9
Game^b^	172	155	90.1
Crops^c^	296	32	10.8
Others^d^	451	106	23.5
N	7,668	972	12.7

^a^River fish, mainly char.

^b^Mainly wild boar (93.0%), pheasant, and rabbit.

^c^Mainly chestnuts (55.4%), beans, and buckwheat.

^d^Mainly wild honey (22.5%) and produced foods combined with fungi.

**Table 2:** Distribution of radiocesium in three items.

Food Type	Season^a^	Number of samples	Median (mini-max) (Bq/kg fresh)		Geometric means ± geometric standard deviations (Bq/kg fresh)	
			^134^Cs	^137^Cs	^134^Cs	^137^Cs
Vegetables	Spring	300	5 (<5–181)	5 (<5–331)	5.8 ± 1.5	6.4 ± 1.8
	Summer	2,186	5 (<5–67)	5 (<5–103)	5.1 ± 1.1	5.1 ± 1.2
	Autumn	1,430	5 (<5–49)	5 (<5–86)	5.1 ± 1.2	5.3 ± 1.3
	Winter	164	5 (<5–63)	5 (<5–206)	5.1 ± 1.2	5.2 ± 1.4
Edible wild plants and fungi	Spring	1,032	20 (<20–1,939)	20 (<20–4,788)	30.2 ± 2.3	38.9 ± 3.0
	Summer	336	20 (<20–1,232)	20 (<20–2,612)	22.2 ± 1.6	24.8 ± 1.9
	Autumn	609	62 (<20–2,754)	149 (<20–8,487)	70.9 ± 3.4	140.3 ± 4.8
	Winter	9	59 (<20–307)	166 (<20–716)	56.1 ± 2.3	130.0 ± 3.2
Fruits	Spring	1	n.d.^b^	n.d.	n.d.	n.d.
	Summer	284	5 (<5–17)	5 (<<5–30)	5.2 ± 1.2	5.9 ± 1.4
	Autumn	329	5 (<5–149)	5 (<5–457)	5.4 ± 1.5	6.2 ± 1.9
	Winter	33	5 (<5–57)	5 (<5–204)	5.7 ± 1.7	7.1 ± 2.2
Fish	All seasons	36	23 (<10–169)	60 (<10–397)	35.7 ± 2.1	60.6 ± 2.6
Game	All seasons	172	210 (<20–5,248)	513 (<20–16,585)	192.1 ± 3.3	460.3 ± 3.8
Crops	All seasons	296	5 (<5–225)	5 (<5–522)	23.2 ± 1.6	27.6 ± 2.1
Others	All seasons	451	20 (<20–584)	20 (<20–1,818)	27.2 ± 1.9	41.4 ± 2.9

^a^From April 2013 to December 2014: Spring is March to May, summer June to August, autumn September to November, and winter from December to January.

^b^n.d.; Not detected.

**Table 3:** Committed Effective Doses from Three Food Types^b^.

Food Type	Seasons^a^	Gender	Age 0–19	20–29	30–39	40–49	50–59	60–69	>70
Vegetables	Spring	Male	2.11–4.34	2.91	2.96	3.48	4.11	3.07	3.58
		Female	2.06–4.26	3.35	2.96	4.12	4.34	5.10	3.29
	Summer	Male	2.79–5.75	4.20	4.99	4.75	5.73	7.08	6.99
		Female	2.50–5.15	4.58	4.99	5.87	5.61	7.61	6.05
	Autumn	Male	1.88–3.87	3.96	2.61	3.38	4.11	5.29	4.09
		Female	1.95–4.02	3.45	3.38	3.42	4.15	5.62	3.81
	Winter	Male	2.28–4.70	4.88	3.73	3.13	5.49	4.69	4.08
		Female	2.06–4.26	4.24	2.94	3.01	4.76	4.54	3.60
Edible wild plants and fungi	Spring	Male	0.25–0.51	0.23	0.41	0.23	0.82	0.58	0.70
		Female	0.50–1.03	0.29	0.41	0.17	0.64	0.70	0.70
	Summer	Male	0.04–0.09	0.12	0.12	0.47	0.47	0.18	0.41
		Female	0.34–0.69	0.41	0.21	0.29	0.47	0.35	0.71
	Autumn	Male	1.44–3.02	1.98	2.55	5.67	5.67	6.80	7.65
		Female	3.09–6.47	9.64	1.70	6.52	6.52	4.25	5.67
	Winter	Male	2.18–4.57	3.58	3.88	5.67	11.94	6.56	6.86
		Female	3.70–7.77	3.28	1.79	4.18	10.15	9.85	5.37
Fruits	Spring	Male	0.55–1.10	0.25	0.48	0.66	0.82	0.96	0.64
		Female	0.59–1.19	0.50	0.48	0.61	0.78	1.15	0.72
	Summer	Male	0.82–1.64	0.80	0.96	1.04	1.15	1.12	1.43
		Female	0.77–1.55	0.85	0.96	0.90	1.33	1.57	1.22
	Autumn	Male	0.71–1.43	0.91	0.43	1.33	1.11	1.65	1.29
		Female	0.80–1.61	0.99	0.77	1.03	1.30	1.12	1.22
	Winter	Male	0.70–1.41	0.49	1.02	0.75	1.36	1.57	1.27
		Female	0.69–1.39	0.64	1.12	1.15	1.32	1.52	1.65

^a^From April 2013 to December 2014: Spring is March to May, summer June to August, autumn September to November, and winter from December to January.

^b^Committed effective doses from vegetables, edible wild plants and mushrooms, and fruits due to radiocesium in Kawauchi Village (μSv/3 months). The range of average intake for vegetables is 179–517 g (minimum – maximum), for edible wild plants and fungi is 1–40 g, and for fruits is 1–178 g based on the information issued by the Environmental Radioactivity Monitoring Center of Fukushima, Fukushima Prefecture, Japan, for all age categories.
